# The influence of low-dose s-ketamine on postoperative delirium and cognitive function in older adults undergoing thoracic surgery

**DOI:** 10.1186/s13019-024-02811-x

**Published:** 2024-06-07

**Authors:** Yujia Wang, Bingqiang Ma, Chaochang Wang, Yingqi Wang, Aijia Liu, Lihua Hang

**Affiliations:** Department of Anesthesiology, Kunshan First People’s Hospital, Kunshan Hospital Affiliated to Jiangsu University, No.566 Qianjin East Road, Kunshan, 215300 China

**Keywords:** Postoperative delirium, Cognitive dysfunction, Older patients, Thoracic surgery, S-ketamine, Neuroprotection, Safety Profile

## Abstract

**Background:**

Postoperative delirium (POD) and cognitive dysfunction (POCD) are common complications following thoracic surgery, particularly in patients aged 65 years and above. These complications can significantly affect recovery and increase healthcare costs. This study investigates the effects of low-dose S-ketamine on reducing POD and POCD in this patient demographic.

**Methods:**

In this retrospective cohort study, medical records of patients aged ≥ 65 years who underwent elective thoracic surgery from January 2019 to August 2023 were reviewed. Patients were categorized into S-ketamine and Control groups based on intraoperative S-ketamine exposure. POD was assessed using the Confusion Assessment Method (CAM), while cognitive function was evaluated using the Montreal Cognitive Assessment (MoCA) at baseline, 1 week, 1 month, and 6 months post-surgery. Intraoperative and postoperative parameters, including hemodynamic stability, blood loss, pain scores, and ICU stay length, were also recorded.

**Results:**

The study comprised 140 participants, with 70 in each group. The S-ketamine group demonstrated a significantly lower incidence of POD at 7 days post-surgery (12.0% vs. 26.7%, *P* < 0.001), and reduced POCD at 1 month (18.7% vs. 36.0%, *P* < 0.05) and 6 months (10.7% vs. 21.3%, *P* < 0.05). The Ketamine group had a significantly higher median MoCA score compared to the Control group both at 1 month (*P* = 0.021) and 6 months (*P* = 0.007). Adverse events, such as infection, bleeding, and respiratory failure, showed no significant differences between the groups, suggesting a safe profile for S-ketamine.

**Conclusion:**

Administering low-dose S-ketamine during thoracic surgery in patients aged 65 years and above significantly reduces the incidence of POD and POCD, highlighting its neuroprotective potential. These findings advocate for the inclusion of S-ketamine in anesthetic protocols to improve postoperative outcomes and reduce healthcare costs in this patient population.

## Introduction

The surgical management of thoracic conditions in older patients is a complex and delicate process, often complicated by postoperative delirium (POD) and postoperative cognitive dysfunction (POCD). According to the DSM-5 definition, delirium is characterized by a disturbance in attention and awareness, often accompanied by additional disturbance in cognition such as memory deficit, disorientation, language disturbance, or perceptual abnormalities [1]. These symptoms develop over a short period, usually fluctuating in severity throughout the day [1]. It is reported that POD usually occurs within 72 h after surgery, affecting approximately 10–50% of older adults undergoing major surgery [2]. Only 4% of older patients with POD fully recover upon discharge, and up to 80% of patients still have residual injuries at or after 6 months [3]. It is associated with increased morbidity, mortality, and healthcare costs [4, 5]. Similarly, POCD, a decline in cognitive function following surgery, can persist for weeks to months, impacting the patient’s quality of life and ability to return to preoperative activities. POCD is most impacted between 3 and 6 months postoperatively, but generally shows improvement over time. The incidence is 29% in the 1 to 3 months postoperatively, decreasing to 14.1% between 3 and 6 months [6]. As the global population ages, the incidences of POD and POCD are expected to rise, underscoring the need for effective preventive strategies.

The pathophysiology of POD and POCD is multifactorial, involving factors such as systemic inflammation, neuroinflammation, and neuronal injury [5]. Inflammation, in particular, plays a pivotal role, with elevated levels of pro-inflammatory cytokines being associated with an increased risk of cognitive complications [7]. S-ketamine, a N-methyl-D-aspartate (NMDA) receptor antagonist, is known for its analgesic properties and has been used in various surgical settings. Recently, it has garnered attention for its potential neuroprotective effects. Its role in inhibiting inflammatory cytokines and reducing neuronal injury makes it a candidate for mitigating POD and POCD [8, 9]. The anti-inflammatory properties of S-ketamine, particularly at low doses, may play a crucial role in preserving cognitive function postoperatively [10, 11]. Given the potential benefits of ketamine, in this study we retrospectively analyzed medical records to evaluate the impact of low-dose S-ketamine on the incidence of POD and POCD in older adults undergoing thoracic surgery.

## Methods

The study was approved by the Ethics Committee of Kunshan Hospital Affiliated to Jiangsu University. All procedures performed in studies involving human participants were in accordance with the 1964 Helsinki declaration and its later amendments or comparable ethical standards. Given its retrospective nature, formal consent was not deemed necessary.

### Study design and participants

**Fig. 1:**
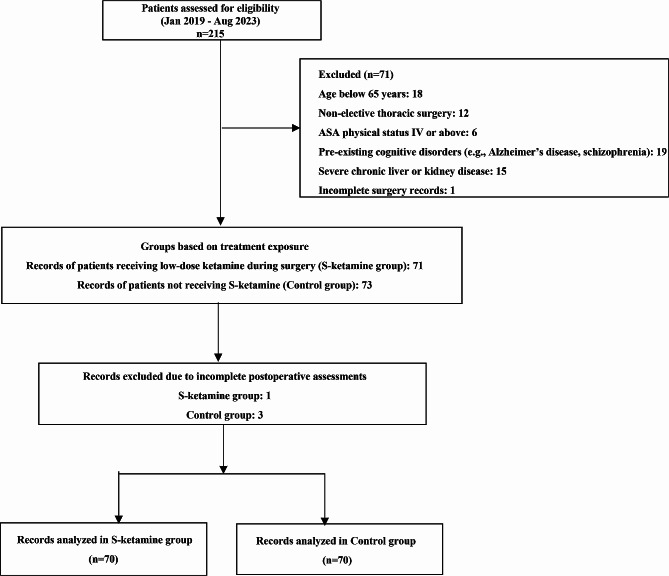
Study flow diagram

This retrospective cohort study reviewed medical records of older adults (aged ≥ 65 years) who underwent thoracic surgery from January 2019 to August 2023 at our hospital (Fig. 1). The inclusion criteria were as follows: (1) patients aged ≥ 65 years; (2) those who underwent elective thoracic surgery under general anesthesia; (3) American Society of Anesthesiologists (ASA) physical status I-III; (4) complete medical records, including Confusion Assessment Method (CAM) and Montreal Cognitive Assessment (MoCA) assessments. We excluded patients with pre-existing cognitive disorders or psychiatric conditions that could affect cognitive assessment (such as Alzheimer’s disease or schizophrenia), severe chronic liver or kidney disease (severe chronic liver disease defined as siagnosed liver cirrhosis or hepatic failure with evidence of synthetic dysfunction (e.g., INR > 1.5, albumin < 30 g/L); severe chronic kidney disease defined as estimated glomerular filtration rate < 30 mL/min/1.73 m², dialysis dependence, or a history of renal transplant rejection), a history of substance abuse including chronic opioid or S-ketamine use, inability to understand or follow postoperative assessment procedures, and patients who underwent emergent or non-elective surgeries.

Patients were divided into two groups based on their exposure to S-ketamine during surgery: the S-Ketamine group which received a continuous intravenous infusion of low-dose S-ketamine (< 0.5 mg/kg/h), and the Control group which did not receive S-ketamine infusion.

### Data collection

The following data will be collected through patient interviews and medical records.

#### Demographic and clinical data

Patient demographic and clinical data were collected including age, sex, weight, height, body mass index (BMI), education levels, comorbidities, history of medications, ASA classification, and type of surgeries.

**Primary outcomes**: The outcome data were collected through standardized protocols to ensure consistency and reliability. Physicians responsible for conducting the CAM and MoCA assessments were trained in cognitive evaluation techniques, ensuring uniformity in scoring. Delirium was assessed using the CAM assessmet after 7 days postoperatively. The CAM consists of four criteria: (1) acute onset and fluctuating course; (2) inattention; (3) disorganized thinking; (4) altered level of consciousness. A patient was considered to have delirium if criteria 1 and 2 are met, along with either criterion 3 or 4. The MoCA scale was applied to assess cognitive function before surgery, as well as 7 days, 1 and 6 months after surgery. The total MoCA score ranges from 0 to 30 points, with higher scores indicating better cognitive function. POCD will be defined as a decline of ≥ 2 points in MoCA score from baseline.

#### Secondary outcomes

Data of depth of anesthesia (BIS), perioperative use of medications, invasive ventilation and organ failures cases were collected. We also monitored an extensive range of intraoperative parameters to provide a detailed assessment of hemodynamic stability. These parameters included heart rate, systolic and diastolic blood pressure, cardiac output (CO), stroke volume (SV), systemic vascular resistance (SVR), oxygen saturation (SpO2), and mean arterial pressure (MAP), Systolic Blood Pressure (SBP), Diastolic Blood Pressure (DBP) and heart rate (HR). Details on intraoperative blood loss and transfusions were also recorded. The visual analogue scale (VAS) score was measured 1 day after surgery with a score of 0–10 indicating no pain to unbearably severe pain. Other outcomes including postoperative complications and stay lengths of ICU were noted.

### Data analysis

Categorical variables were expressed as numbers (percentages) and analyzed using chi-square or Fisher’s exact test. The Shapiro–Wilk method was used to test the normality of the continuous data. Normally distributed measures were expressed as mean ± standard deviation (SD), while non-normally distributed measures were expressed as median (interquartile range). t-test or Mann-Whitney U test was performed for between-group comparisons, and Wilcoxon test was used for within-group comparisons. Data analysis was performed using SPSS software version 26.0, with a P value < 0.05 (two-tailed) considered statistically significant.

## Results

### Patient characteristics

This study incorporated 140 participants, with 70 patients each in the S-ketamine and control groups. Age, gender, weight, height, and body mass index distributions were similar between the groups. Additionally, the incidence rates of hypertension, diabetes, heart disease, and medication usage (ACE inhibitors, β-blockers, statins) were comparable in both groups (Table 1).

**Table 1:** Baseline characteristics of participants in the s-ketamine and control groups

	S-ketamine Group (*n* = 70)	Control Group (*n* = 70)	*P*
**Age (years)**	77.71 ± 7.57	76.90 ± 7.99	0.537
**Sex**			
Female	33 (47.14%)	38 (54.29%)	0.398
Male	37 (52.86%)	32(45.71%)	
**Weight (kg)**	70.54 ± 11.84	71.63 ± 10.44	0.566
**Height (cm)**	165.47 ± 9.74	166.65 ± 10.89	0.498
**BMI (kg/m** ^**2**^ **)**	26.01 ± 5.26	25.94 ± 4.18	0.934
**Level of education**			
≤primary school	15 (21.43%)	18 (25.71%)	0.394
Middle school or high school	20 (28.57%)	25 (35.71%)	
≥above high school	35 (50.00%)	27 (38.57%)	
**Comorbidities**			
Hypertension	15(21.43%)	17 (24.29%)	0.840
Diabetes	22(31.43%)	17 (24.29%)	0.451
Heart disease	17(24.29%)	18 (25.71%)	1.000
None	16(22.86%)	18 (25.71%)	0.844
**History of medications**			
ACEI	16 (22.86%)	15 (21.43%)	0.793
Beta-blockers	21 (30.00%)	17 (24.29%)	
Statins	19 (27.14%)	24 (34.29%)	
None	14 (20.00%)	14 (20.00%)	
**ASA**			0.797
I	10 (14.29%)	12 (17.14%)	
II	35 (50.00%)	32 (45.71%)	
III	25 (35.71%)	26 (37.14%)	
**MoCA score (baseline)**	24.0 (21.0, 26.0)	23.0 (21.0, 25.0)	0.272
**Type of surgery**			
Lobectomy	30 (42.86%)	28 (40.00%)	0.689
Segmentectomy	15 (21.43%)	17 (24.29%)	
Thoracoscopic exploration	10 (14.29%)	12 (17.14%)	
Mediastinoscopy	8 (11.43%)	7 (10.00%)	
Pleurectomy	7 (10.00%)	6 (8.57%)	

Note: ASA, American Society of Anesthesiologists; MoCA, Montreal Cognitive Assessment; BMI, Body Mass Index; ACEI, Angiotensin-Converting Enzyme Inhibitors; P-value < 0.05 indicates statistical significance

### Intraoperative and postoperative patient characteristics

As shown in Table 2, the duration of surgery and anesthesia was similar across both groups. During surgery, BIS values and perioperative use of medications were consistent. HR, SBP, DBP, blood loss, blood transfusion volumes, CO, SV, SVR, and SpO_2_, invasive ventilation and organ failures cases showed no significant differences between groups. Notably, postoperative data indicated a marginally lower VAS score in the S-ketamine group compared to the control group, although this difference was not statistically significant (*P* = 0.082). Additionally, the length of ICU stay was similar between the groups.

**Table 2:** Comparison of intraoperative and postoperative parameters between S-ketamine and control groups in thoracic surgery patients

	S-ketamine Group (*n* = 70)	Control Group (*n* = 70)	*P*	
**Duration of surgery (h)**	3.00 ± 1.05	3.10 ± 1.30	0.627	
**Duration of anesthesia (h)**	3.49 ± 1.43	3.49 ± 1.35	0.976	
**Depth of Anesthesia (BIS)**	49.10 ± 6.56	49.37 ± 6.80	0.992	
**Use of Propofol, n (%)**	63 (90.00%)	61 (87.14%)	0.812	
**Use of Remifentanil, n (%)**	55 (78.57%)	52 (74.29%)	0.881	
**Use of Sufentanil, n (%)**	58 (82.86%)	46 (65.71%)		
**Use of Dexamethasone, n (%)**	32 (45.71%)	38 (54.29%)	0.398	
**Intraoperative data**				
HR (bpm)	89.56 ± 12.95	89.26 ± 13.09	0.892	
SBP (mmHg)	124.41 ± 13.93	125.61 ± 14.92	0.624	
DBP (mmHg)	76.24 ± 9.02	75.84 ± 8.59	0.789	
MAP (mm Hg)	83.0 (78.25, 89.75)	85.0 (79.25, 89.75)	0.541	
CO (L/min)	4.60 (4.10, 5.60)	4.90 (4.20, 5.68)	0.423	
SV (mL)	80.50 (71.25, 93.75)	79.00 (71.00, 87.00)	0.143	
SVR (dyn·s/cm)	1255.00 (1110.00, 1477.50)	1225.00 (1070.00, 1387.50)	0.323	
SpO_2_ (%)	95.55 (93.85, 98.18)	95.40 (93.70, 97.95)	0.854	
Amount of blood loss (ml)	296.00 (141.50, 380.25)	298.50 (168.75, 384.75)	0.717	
Volume of blood transfusion (ml)	133.00 (45.75, 219.25)	129.00 (69.25, 214.75)	0.881	
Invasive ventilation	19 (27.14%)	25 (35.71%)	0.363	
**Postoperative data**				
VAS score	4.39 ± 3.20	5.23 ± 2.46	0.082	
Organ Failures	13 (18.57%)	18 (25.71%)	0.416	
ICULOS (days)	4.00 (2.00, 5.00)	4.00 (3.00, 5.00)	0.249	

Note: bpm, beats per minute; mmHg, millimeters of mercury; ml, milliliters; mg, milligrams; VAS, Visual Analog Scale; ICULOS, Intensive Care Unit Length of Stay; HR, Heart Rate; SBP, Systolic Blood Pressure; DBP, Diastolic Blood Pressure. BIS, Bispectral Index. P values are reported for the comparison between the Ketamine and Control groups

### Postoperative delirium and cognitive dysfunction between groups

Assessing the incidence of POD, results revealed that the occurrence was significantly lower in the S-ketamine group compared to the control group after 7 days (12.0% vs. 26.7%, *P* < 0.001). However, there was no significant difference in incidence of POCD within 7 days after surgery between the two groups. Of note, the incidence of POCD at 1 (18.7% vs. 36.0%, *P* < 0.05) and 6 months (10.7% vs. 21.3%, *P* < 0.05) after surgery was also significantly lower in the S-ketamine group compared with the control group. The Ketamine group had a significantly higher median MoCA score compared to the Control group both at 1 month (*P* = 0.021) and 6 months (*P* = 0.007) (Table 3).

**Table 3:** Primary outcomes of postoperative delirium and cognitive dysfunction

	Ketamine Group (*n* = 70)	Control Group (*n* = 70)	*P*
**Incidence of postoperative delirium after 7 days**	8 (12.0%)	19 (26.7%)	< 0.001^*^
**Incidence of postoperative cognitive dysfunction at 7 days**	11 (15.71%)	13 (18.57%)	0.198
**Incidence of postoperative cognitive dysfunction at 1 month**	13 (18.7%)	25 (36.0%)	< 0.05^*^
**Incidence of postoperative cognitive dysfunction at 6 months**	7 (10.7%)	15 (21.3%)	< 0.05^*^
**MoCA score after 1 month**	23.5 (22.0, 25.75)	22.0 (20.5, 24.0)	0.021*
**MoCA score after 6 months**	26.0 (23.0, 28.0)	24.0 (21.0, 26.0)	0.007*

Notes: MoCA, Montreal Cognitive Assessment; MoCA score is presented as median (25th percentile, 75th percentile). P-values indicate the level of statistical significance between the Ketamine and Control groups with *P* < 0.05 considered significant

### Adverse events

As illustrated in Table 4, there were no significant differences in the rates of infection, bleeding, and respiratory failure between the two groups. The majority of patients in both groups did not experience any of these adverse events.

**Table 4:** Incidence of treatment-emergent adverse events and serious adverse events

	Ketamine Group (*n* = 70)	Control Group (*n* = 70)	*P*
**Infection**	9 (12.86%)	11 (15.71%)	0.575
**Bleeding**	7 (10.00%)	12 (17.14%)	0.210
**Respiratory failure**	9 (12.86%)	11 (15.71%)	0.575
**None**	43 (61.43%)	36 (51.43%)	0.215

Note: Data presented as number of patients with events (percentage of total). P-values calculated using the Fisher’s exact test where applicable, reflecting a comparison of the incidence rates between the two groups. Differences were not considered statistically significant at the *P* < 0.05 threshold. Abbreviations: n, number of patients

## Discussion

In this study, low-dose S-ketamine’s impact on POD and POCD in older adults undergoing thoracic surgery was explored. It is widely acknowledged that POD, a frequent and distressing complication following thoracic surgery, is particularly prevalent in the elderly, with incidence rates oscillating between 4% and 60% [12, 13]. Ketamine, a non-competitive N-Methyl-D-Aspartate (NMDA) receptor [14] antagonist, is pharmacologically rationalized as an effective agent in reducing POD [15]. S-Ketamine is the D-isomer of Ketamine, also known as “esketamine”. Our findings revealed that low-dose S-ketamine significantly reduced the occurrence of POD and ameliorated POCD in elderly patients undergoing thoracic surgery, implying potential neuroprotective attributes of ketamine. However, these results contrast with a recent meta-analysis by Viderman et al. [3], which found no statistically significant difference in incidences of POD between the ketamine and control groups. Such discrepancies may stem from differences in surgical populations. Our study focused on the high-risk subgroup of older adults undergoing thoracic surgery, which inherently carries a higher risk of postoperative neurocognitive disorders due to factors like advanced age, comorbidities, and surgical stress. The neuroprotective effects of S-ketamine could be more pronounced in this subgroup due to heightened vulnerability. Moreover, our dosing regimen of low-dose S-ketamine may have resulted in pharmacological effects that differ from other regimens evaluated in Viderman et al.‘s analysis. The research findings by Lu and colleagues were found to be consistent with ours. It was observed that S-ketamine could be employed for the prevention of post-anesthetic delirium in elderly thoracic surgery patients [16]. However, it was suggested by Bornemann et al. that a low dose of S-ketamine could be considered as a useful and low-risk component for perioperative balanced analgesia. The administration of low-dose S-ketamine (0.25 mg/kg bolus) was also found to exacerbate patient delirium [17]. The study conducted by Yang and colleagues presented a contrasting perspective. Their findings indicated that administering S-ketamine (0.2 mg/kg) at the end of anesthesia effectively reduced the incidence and severity of delirium in preschool children undergoing tonsillectomy and/or adenoidectomy. Importantly, this intervention did not prolong extubation time or increase adverse events [18]. The dosage utilized by Yang and colleagues was lower than that employed by Bornemann et al. These results suggest that different doses of S-ketamine may lead to distinct pharmacological effects, which may be associated with patient tolerance and sensitivity. In our study, the limitation arises from a relatively small sample size, and we did not further stratify the doses of S-ketamine. This limitation will be addressed in future research endeavors with larger sample sizes and a more detailed examination of S-ketamine dosages.

Additionally, in older patients, minor changes in postoperative neurological function have been more frequently observed [19]. POCD, another potential complication of surgical interventions, has been found to be more prevalent among older adults and those undergoing cardiac surgery [20]. It was previously shown that sub-anesthetic doses of S-ketamine (10 mg/kg) improve POCD by inhibiting hippocampal astrocytosis in a mouse model of post-stroke chronic stress [21]. In a comparable manner, in another randomized controlled study, subanesthetic doses of S-ketamine (0.15 mg/kg) were found to potentially decrease the occurrence of early delayed neurocognitive recovery in postoperative stages of older adults, thereby enhancing early postoperative cognitive function. This effect might be linked to the anti-neuroinflammatory properties of S-ketamine [22]. Our research demonstrated that compared to placebo, S-ketamine improved POCD at one month and even at six months post-surgery. Taken together with our results, the dose of s -ketamine does not appear to affect its efficacy in improving cognitive function. Another meta-analysis showed no significant difference in the incidence of POCD within 7 days for intraoperative low-dose S-ketamine compared to the control group [23]. This is consistent with our results. This may mean that it takes longer for S-ketamine to be effective in adults.

Our study has several limitations that warrant further discussion. First, the retrospective design of our study introduces potential biases in patient selection and data collection, which could influence the outcomes observed. Secondly, the smaller sample size may introduce a degree of randomness, necessitating larger cohorts to bolster the credibility of the conclusions. Thirdly, several potentially influential factors were not explored in this study, such as lung protective strategies, which may influence cognitive outcomes post-surgery. Although the MoCA scale was chosen as the primary cognitive assessment tool, it does not provide the comprehensive assessment required for a nuanced understanding of POCD. The more comprehensive cognitive assessment methods rather than use a single method could yield more accurate results. Moreover, we will further analyze the relationship between POD and POCD in future studies. We recommend cautious interpretation of our findings, future research should focus on randomized controlled trials to confirm these findings and refine S-ketamine dosages and administration protocols to maximize benefits while minimizing risks.

## Conclusion

Our study’s results emphasized the potential advantages of employing low-dose S-ketamine in geriatric thoracic surgery patients, especially in reducing POD and enhancing cognitive function. Despite previous research raising concerns, our findings lend support to the use of S-ketamine as a safe and efficacious adjunctive anesthetic agent. These findings hold substantial clinical significance, potentially contributing to the enhancement of postoperative recovery quality for older adults.

## Data Availability

No datasets were generated or analysed during the current study.
